# Posttraumatic stress disorder and health: a preliminary study of group differences in health and health behaviors

**DOI:** 10.1186/1744-859X-12-30

**Published:** 2013-09-26

**Authors:** Kathryn M Godfrey, Laurie A Lindamer, Sheeva Mostoufi, Niloofar Afari

**Affiliations:** 1 San Diego State University/University of California, San Diego Joint Doctoral Program in Clinical Psychology, 6363 Alvarado Ct, San Diego, CA 92120, USA; 2 VA Center of Excellence for Stress and Mental Health and VA San Diego Healthcare System, 3350 La Jolla Village Drive, San Diego, CA 92161, USA; 3 Department of Psychiatry, University of California, San Diego, 9500 Gilman Dr. #0737, La Jolla, CA 92093, USA

**Keywords:** Alcohol use, Behavioral medicine, Body mass index, Diet, Exercise, Posttraumatic stress

## Abstract

**Background:**

Individuals with posttraumatic stress disorder (PTSD) are more likely to undertake harmful health behaviors like substance use. Less is known about the association of PTSD with healthful behaviors such as healthy diet and exercise. The purpose of this study was to examine differences across physical health indicators and health behaviors in individuals with and without PTSD.

**Methods:**

A cross-sectional, case–control study of health indicators and self-reported health behaviors in a community and military veteran sample was used.

**Results:**

Based on a structured psychiatric interview, 25 participants had PTSD, and the remaining 55 without PTSD served as the comparison group. Participants were 40 years old on average and 45% were female. Multivariate analysis of variance analyses revealed that participants with PTSD had significantly higher body mass index (*p* = 0.004), had more alcohol use (*p* = 0.007), and reported fewer minutes of vigorous exercise (*p* = 0.020) than those without PTSD. Chi-square analysis of diet content and eating behavior constructs found that individuals with PTSD ate fewer fruits (*p* = 0.035) and had more guilt after overeating (*p* = 0.006).

**Conclusions:**

These findings replicate prior research on the link between PTSD and negative health outcomes and engagement in harmful health behaviors and highlight the need for further examination of the association between PTSD and other health behaviors like diet content, eating behaviors, and exercise.

## Background

The experience of trauma is common in the USA as about 60% of men and 51% of women disclose at least one lifetime exposure to a traumatic event [1]. While most individuals recover from trauma-related symptoms, such as intrusive thoughts, numbing, and hyperarousal, some will develop posttraumatic stress disorder (PTSD), a persistence of symptoms severe enough to impair daily functioning. Current estimates for lifetime prevalence rates of PTSD in the general US population are between 6.4% and 7.8% [1-3], with lifetime rates of up to 31% in US military populations and equivalent or slightly lower prevalence rates for veterans from the UK [4].

PTSD not only has psychiatric implications but may also have a significant effect on health, further impairing functioning and quality of life. Individuals with PTSD have poorer overall health, lower health-related quality of life, and increased work absenteeism compared to people without PTSD [5-8]. PTSD appears to negatively affect physical health functioning even more than other mental disorders like panic disorder, generalized anxiety disorder, and major depressive disorder [9], and PTSD negatively impacts physical health to a greater extent than commonly comorbid conditions, like substance use disorders [10]. Individuals with PTSD have increased rates of chronic health conditions like obesity [11-13], diabetes [14], hypertension [15,16], heart disease [17,18], and metabolic and cardiovascular diseases [17,19,20] relative to those without PTSD. Given the broad range of physical health problems associated with PTSD, it could be postulated that there are a number of general negative health-related behaviors underlying its detrimental effect on health.

There is a large body of research examining the link between PTSD and harmful or negative health behaviors, such as tobacco use [21,22] and alcohol abuse and dependence [1,23]. Even years after exposure to traumatic events, increased symptoms of PTSD are associated with worse general health and increased rates of smoking and at risk drinking [24]. There are several reports in the literature demonstrating that PTSD is associated with negative health outcomes, such as unhealthy weight and unfavorable high waist-hip ratios, and with poor indicators of health behaviors like low fitness levels and inadequate nutritional status [11,12,25], all of which are known to contribute to the development of physical conditions.

There has been less research examining PTSD and healthful behaviors directly, but the initial findings suggest that individuals with PTSD may engage less in these positive health behaviors. For example, one study suggests that veterans with PTSD are less likely to engage in physical activity and exercise [26]. Recently, a study of individuals with cardiovascular disease found that individuals with PTSD were less physically active than those without PTSD [22]. A large, prospective study of veterans found that vigorous exercise was associated with decreased odds of PTSD symptoms [27]. Exercise has also been suggested as a protective factor against developing fibromyalgia in men with PTSD [28]. The potential for exercise to impact PTSD symptoms in clinical settings has been noted in a recent randomized controlled trial proposal examining the added effect of an exercise program to PTSD treatment as usual [29]. More research is needed to provide further evidence of how individuals with PTSD exercise and the relationship between exercise and PTSD. Other researchers have found a relationship between PTSD and eating behaviors, specifically, a high comorbidity of PTSD and eating disorders [30,31]. However, less is known about how PTSD may impact diet and eating behaviors in a non-eating disorder population. No research has yet examined how individuals with PTSD compare to individuals without PTSD across non-pathological diet and eating behaviors, which may be essential to overall health.

Theoretical models of the relationship between PTSD and health that include health behaviors often present only negative health behaviors like substance use [19], omitting the potentially protective factors like exercise and healthy diet. A recent review of the specific immunological mechanisms identified both diet and exercise as possible factors in the model of PTSD and health because of their known impact on inflammation [32]. Diet and eating behaviors might also be negative health behaviors in which individuals with PTSD engage. A study of young women found an association between PSTD and increased soda and fast food consumption in addition to disordered eating behaviors like skipping meals and self-induced vomiting [33]. However, further evidence is needed before health behaviors like diet and exercise can be integrated into the theoretical models of PTSD and health. With further empirical support for these models and translation into clinical practice, identifying both positive and negative health behaviors associated with PTSD could aid in the development of behavioral interventions that might prevent or mitigate the poor health outcomes seen in those with PTSD.

The primary aims of this study were (1) to replicate findings that individuals with PTSD have worse outcomes across physical health indicators in a relatively healthy sample free from significant chronic health conditions, (2) to further examine whether individuals with PTSD engage more in negative health behaviors like alcohol use and less in positive health behaviors like exercise, and (3) to preliminarily examine differences in a brief assessment of eating behaviors and diet content in individuals with PTSD compared to non-PTSD controls. It was hypothesized that individuals with PTSD would have poorer physical health, be more likely than controls to engage in negative health behaviors like substance use, and be less likely to engage in positive health behaviors like exercise. It was also hypothesized that individuals with PTSD would report more unhealthy eating behaviors and diet content than controls.

## Methods

### Participants

Participants with PTSD and non-PTSD controls were community members and US military veterans recruited through community flyers and advertisements, physicians, and clinics at the Veterans Affairs San Diego Healthcare System (VASDHS) and the University of California, San Diego (UCSD). Participants' eligibility was assessed by telephone screening. To ensure the sample did not consist of chronically ill participants, exclusion criteria consisted of major medical illnesses (e.g., cancer, kidney failure, stroke, cardiovascular disease, seizure), physical impairments that would preclude participation (e.g., blindness, deafness), schizophrenia, bipolar disorder, personality disorders (e.g., borderline and antisocial), eating disorders, current substance abuse or dependence, and a history of chronic pain lasting more than 6 months. Of the 267 individuals who were contacted for a telephone screen, 145 were initially eligible, and of those, 80 had confirmed PTSD or were controls without PTSD or anxiety disorders and were included in the study. The study was approved by the Institutional Review Board of UCSD and by the Research and Development Committee of VASDHS. All participants gave written informed consent and received a small payment ($45) for their time and effort. All authors had access to the data.

### Measures

#### Psychiatric diagnosis

The Composite International Diagnostic Interview (CIDI) anxiety disorders module was used to confirm PTSD and control group placement [34]. The CIDI was administered by bachelor's-level research assistants who received observation- and practice-based training with experienced and trained assessors and ongoing supervision by a licensed clinical psychologist. The CIDI is a widely used diagnostic interview with population-based samples and has acceptable psychometric properties [35]; the PTSD module has adequate agreement with clinician ratings [36].

#### Physical health indicators

Physical health indicators were taken during the participants' single visit to the lab. Systolic and diastolic blood pressure and resting heart rate were measured using a commercially available blood pressure monitor (Omron HEM-712C, Omron Healthcare, Inc., Bannockburn, IL, USA). A research assistant measured height with a stadiometer, collected hip and waist measurements according to standard procedures [37], and obtained participants' weight using a standard scale. Body mass index (BMI) was calculated from height and weight using the standard formula (Weight (kg)/Height (m)^2^). Hip-to-waist ratio was calculated from hip size and waist size.

#### Health behaviors

The Alcohol Use Disorders Identification Test (AUDIT) was used to measure alcohol use [38]. AUDIT is a 10-item self-report instrument created to assess hazardous and harmful alcohol consumption with higher scores indicating more hazardous or harmful drinking. This measure has acceptable psychometric properties [38] and acceptable internal consistency in this study (Cronbach's *α* = 0.79).

The Short Form of the International Physical Activity Questionnaire (IPAQ-SF) assessed physical activity [39]. IPAQ-SF was scored according to standard procedures to compute the continuous metabolic equivalent of task minutes per week (METmpw) variables for vigorous, moderate, and walking activities [40]. Scoring and computation of the IPAQ-SF variables was done by researchers blind to participant diagnosis and research hypotheses. The IPAQ-SF has acceptable psychometric properties [39].

At the time of the study, no brief measures were available assessing all of the particular dietary content and eating behaviors of interest. Therefore, we generated several questions to capture the diet and eating behavior constructs relevant to this preliminary study. There were five questions about dietary contents: consumption of fruits, vegetables, soda, caffeinated beverages, and fast food. These questions expanded upon similar research examining soda and fast food consumption in young women with PTSD [33]. Participants were asked to report how many servings they consumed on a typical day during the past 4 weeks. These questions were presented to participants with four response choices (none, 1–2, 3–4, 5 or more) that were later collapsed into two response choices (none to 2, 3 or more) to ensure adequate cell sizes for analysis. For example, the original responses to the consumption of fruit question had only two participants (one control, one with PTSD) endorsing ‘none’ and only eight participants (seven control, one with PTSD) endorsing five or more with the majority of participants selecting the middle (1–2, 3–4) response choices. The four eating behavior questions asked about (1) consciousness of eating, (2) eating sensibly in front of others and splurging alone, (3) giving too much time and thought to food, and (4) having feelings of guilt after overeating. These items were of interest because of the literature examining the roles of mindful eating, overeating, and binge eating in obesity and overweight [41-43]. All eating behavior questions were presented to participants with four response options which were also dichotomized to ensure sufficient cell size for analysis. The four responses for the consciousness of eating question (not at all, slightly, moderately, very much) were grouped into two responses (not at all to slightly, moderately to very much). Similarly, the three other eating behavior questions presented four responses (never, rarely, often, always) that were dichotomized (never to rarely, often to always) for analytical purposes. No psychometric properties were derived for these questions as they were not intended to represent or create a new scale of diet content and eating behaviors, and each question only targets one construct of interest. These questions were derived for this preliminary study to direct future research into PTSD and eating behaviors and diet content.

### Statistical analyses

Sociodemographic characteristics were compared between groups using Pearson chi-square (*χ*^2^) tests for categorical data and one-way between-subjects analysis of variance (ANOVA) for continuous measures. Group comparisons along continuous measures were performed with a between-subjects multivariate analysis of variance (MANOVA). Group differences across physical health were examined with a MANOVA performed on the five physical health indicators: BMI, waist-hip ratio, systolic blood pressure, diastolic blood pressure, and resting heart rate. Differences in continuous health behaviors were examined with a MANOVA containing four health behaviors: alcohol use, vigorous exercise, moderate exercise, and walking. Using an alpha level of 0.001 to evaluate homogeneity assumptions for the two MANOVAs, Box's *M* test of homogeneity of covariance (*p* ≥ 0.001) and Levene's homogeneity test (all *p*'s ≥ 0.001) were not statistically significant. Differences in diet content and eating behaviors were examined with *χ*^2^ tests. Significance level was set at <0.05. Statistical analyses were conducted using Statistical Package for Social Sciences version 20 (IBM, Armonk, NY, USA).

## Results

Table 1 presents the sociodemographic characteristics of the entire sample and by group. Participants were mainly non-veteran males and females with an average age of 39.9 years. Sixty percent of study participants self-identified as Caucasian, 21% reported being Hispanic or Latino, and 19% self-identified as Black or African American. Approximately 51% were single and never married, 43% had a college degree or higher, and 44% reported a yearly income of $30,000 or higher. PTSD (*n* = 25) and control (*n* = 55) groups did not differ across veteran status, gender, ethnicity, marital status, income, or age. Participants in the PTSD group, however, had significantly lower education levels (*χ*^2^ (6, *N* = 80) = 19.24, *p* = 0.004).

**Table 1:** Sociodemographic characteristics of the entire sample and by group

	**Total**	**Control**	**PTSD**
	**(*N *= 80)**	**(*n *= 55)**	**(*n *= 25)**
Non-veteran (%)	93	95	88
Female (%)	45	44	48
Ethnicity/race (%)			
White	60	60	60
Black/African American	19	16	24
Hispanic/Latino	21	16	32
Education (%)			
Eleventh grade or less	4	2	8
High school graduate	6	2	16
Some college	36	33	44
Technical/vocational school graduate	11	9	16
Bachelor's degree	28	38	4
Graduate or professional degree	15	16	12
Marital status (%)			
Single, never married	51	55	44
Divorced	19	15	24
Married	15	16	12
Income (%)			
<$20,000	41	33	60
$20,000–$39,000	30	35	20
$40,000–$59,000	11	13	8
$60,000–$80,000	6	5	8
>$80,000	10	13	4
Age: *M* (SD)	39.9 (13.5)	39.3 (13.6)	41.4 (13.3)

Table 2 presents the raw means, standard deviations, and results of the two MANOVAs for all continuous outcome variables for the entire sample and by group. Taken together, the sample was overweight, with average systolic and diastolic blood pressure and heart rate within normal limits. The average score on the AUDIT was consistent with moderate alcohol use. The sample reported substantial levels of vigorous and moderate physical activity as well as walking per week. For the MANOVA analyses using Wilk's criterion (*λ*) as the omnibus test statistic, the combined physical health indicator dependent variable had a significant main effect for group—*F*(5, 74) = 3.257, *p* = 0.010, and partial *η*^2^ = 0.180. Individuals with PTSD had significantly higher BMI—*F*(1, 78) = 8.920 and *p* = 0.004—than the participants without PTSD. The main effect of group was also significant for the health behavior variables—*F*(4, 70) = 5.157, *p* = 0.001, and partial *η*^2^ = 0.228. Participants with PTSD had increased alcohol use—*F*(1, 73) = 124.522 and *p* = 0.007—and less vigorous physical activity—*F*(1, 73) = 5.685 and *p* = 0.020—compared to individuals without PTSD. There were no other physical health or physical activity differences between the PTSD and control group, but the difference on resting heart rate approached significance—*F*(1, 78) = 3.198 and *p* = 0.078.

**Table 2:** Means, standard deviations, and results of MANOVA models for all continuous outcome variables

	**Total**	**Control**	**PTSD**	***p *value**	**Partial *η***^**2**^
	**(*N *= 80)**	**(*n *= 55)**	**(*n *= 25)**		
Physical health indicators: *M* (SD)				*0.010*	*0.180*
BMI	28.28 (6.75)	26.83 (5.52)	31.47 (8.13)	*0.004*	*0.103*
Waist-to-hip ratio	0.90 (0.09)	0.90 (0.08)	0.90 (0.10)	0.999	<0.001
Systolic BP	127.51 (19.43)	128.25 (21.25)	125.88 (14.93)	0.616	0.003
Diastolic BP	75.89 (12.62)	75.76 (12.89)	76.16 (12.25)	0.897	<0.001
Resting HR	79.25 (14.73)	77.29 (14.06)	83.56 (15.54)	0.078	0.039
Health behaviors: *M* (SD)				*0.001*	*0.228*
AUDIT	3.06 (4.12)	2.21 (2.87)	4.96 (5.66)	*0.007*	*0.097*
Vigorous exercise	1,461 (1,911)	1,791 (2,018)	733 (1,433)	*0.020*	*0.072*
Moderate exercise	989 (1,291)	883 (1,148)	1,218 (1,556)	0.261	0.017
Walking	1,506 (1,304)	1,572 (1,351)	1,364 (1,210)	0.516	0.006

BMI is body mass index in units of kilograms divided by meters squared (kg/m^2^). BP is blood pressure in units of millimeters of mercury (mmHg). HR is heart rate in units of beats per minute (bpm). AUDIT is the Alcohol Use Disorders Identification Test [37]. Exercise variables are from the Short Form of the International Physical Activity Questionnaire (IPAQ-SF) [38] in units of metabolic equivalent of task minutes per week (METmpw). Two MANOVA models were performed: one with health indicators and one with health behaviors. Italics highlight values reaching statistical significance (*α* = 0.05).

Table 3 presents the results of the diet content and eating behavior questions and the findings of group differences. PTSD participants reported eating fewer fruits—*χ*^2^ (3, *N* = 80) = 10.10 and *p* = 0.02—but there were no group differences in the reported consumption of vegetables, soda, caffeine, or fast food. In terms of eating behaviors, individuals with PTSD reported more guilt after overeating—*χ*^2^ (3, *N* = 80) = 9.66 and *p* = 0.022. There were no group differences in splurging alone, consciousness of eating, or thoughts about food.

**Table 3:** Group differences in diet content and eating behavior

	**Total (*N *= 80)**		**Control (*n *= 55)**		**PTSD (*n *= 25)**		
Diet content	0–2	3 or more	0–2	3 or more	0–2	3 or more	*p*
Fruits (%)	59	41	51	49	76	24	*0.035*
Vegetables (%)	63	37	58	42	72	28	0.237
Soda (%)	85	15	85	15	84	16	0.866
Caffeine (%)	75	25	78	22	68	32	0.330
Fast food (%)	81	19	80	20	84	16	0.671
Eating behavior	Never to rarely	Often to always	Never to rarely	Often to always	Never to rarely	Often to always	*p*
Splurge alone (%)	76	24	82	18	64	36	0.083
Too much time and thoughts to food (%)	68	32	65	35	72	28	0.562
Guilt after overeating (%)	73	27	82	18	52	48	*0.006*
	Not at all to slightly	Moderately to very much	Not at all to slightly	Moderately to very much	Not at all to slightly	Moderately to very much	*p*
Conscious of eating (%)	16	84	13	87	24	76	0.205

Pearson chi-square tests were performed for all diet content and eating behavior measures. Italics highlight values reaching statistical significance (*α* = 0.05).

## Discussion

This study examined physical health indicators and both unhealthy and healthful behaviors in those with and without PTSD. Individuals with PTSD had higher BMI, engaged more in negative health behaviors as indicated by higher AUDIT scores and more guilt after overeating, and performed fewer positive health behaviors such as vigorous exercise and eating fruits compared to individuals without PTSD.

We replicated findings [11,19,25] that individuals with PTSD had higher BMI than controls. However, there were no significant differences between those with and without PTSD on several other physical health indicators, such as waist-hip ratio, blood pressure, or heart rate, findings that are not consistent with previous studies [19,25]. One previous study [25] found that PTSD symptoms in women had a significant relationship with waist-hip ratio, but did not directly compare waist-hip ratio values between groups with and without PTSD as done in the current study. The non-significant group differences in physical health indicators in the present study may be due to different sample characteristics like inclusion of male participants and exclusion for certain serious health conditions like cardiovascular disease.

Individuals with PTSD reported more alcohol use and less vigorous exercise compared to individuals without PTSD. No differences were found between groups in moderate exercise or walking. Moderate exercise was less likely to be endorsed by participants in either group, which may be a result of the particular exercise measure used in this study. The IPAQ-SF provides examples of moderate exercise (‘carrying light loads, bicycling at a regular pace, or doubles tennis’) that may not be common forms of moderate exercise in this sample. With respect to walking, the present sample may be too young and/or too healthy overall for differences in walking ability or activity to be present. Therefore, the vigorous exercise variable (‘activities like heavy lifting, digging, aerobics, or fast bicycling’) may have been the best measure of physical activity in this sample. The present findings provide further evidence that individuals with PTSD may engage in more negative health behavior, such as consuming alcohol [1,23], and less in positive health behaviors, like exercise [26,27].

Our preliminary finding on diet content suggests that individuals with PTSD eat fewer fruits per day, relative to those without PTSD. Unlike the one previous study that examined diet and disordered eating behaviors in young women with PTSD [33], however, we did not find group differences in soda or fast food consumption, likely due to different sample characteristics. One's dietary content may have implications for general health. For example, consumption of fruits could protect against many health conditions like cancer, coronary heart disease, diabetes, and obesity [44,45]. Alternately, increased consumption of fast food and soda may lead to a greater burden of conditions such as obesity and diabetes [46,47]. Clearly, more research is needed with a larger and more diverse sample to fully examine the scope of dietary content in PTSD and its impact on both physical and mental health. Specifically, future research can examine whether consuming fewer servings of fruit could be linked with present or future poor health in PTSD as has been found in other populations [48].

We also found that the group with PTSD reported more guilt after overeating, which could be capturing both the affective component of overeating (e.g., shame, regret, guilt) and/or the severity of overeating behavior. Individuals with PTSD often report experiencing guilt or shame as a result of their trauma [49,50], feelings which could generalize to aspects of their day-to-day lives, like eating behaviors. Overeating as a means of emotional regulation or self-medication is also in line with the literature linking PTSD and substance use as there are many shared features between addictions and overeating [33,51,52]. Further, the majority of both women and men diagnosed with eating disorders that have binging components (bulimia nervosa and binge eating disorder) have a history of interpersonal trauma [30], suggesting that trauma exposure may be a risk factor for disordered eating behaviors like binging or overeating. Future research can further examine the potentially complex relationship between the emotional sequelae of trauma exposure, diet content, eating behaviors, and health outcomes in PTSD.

The preliminary findings from this study highlight many directions for future research. Altogether, our findings suggest that individuals with PTSD may have decreased physical health and engage in more negative and less positive health behaviors, which may increase the burden of psychological symptoms. Assessing and treating comorbid substance use is already part of most PTSD treatment plans, but individuals with PTSD may benefit from treatments that examine health behaviors more broadly by including diet and exercise. Studies examining the mediators and mechanisms of these associations might also provide insights for clinical approaches. However, given the novel, preliminary nature of this study, further investigation with larger samples is warranted to create better models of PTSD and health and to establish clinical recommendations. Replication studies would benefit from using varied assessment methodology such as food diaries or mobile applications to capture eating behavior using ecological momentary assessment and pedometers or accelerometers to assess physical activity without relying on self-report.

This study had several limitations. The data are retrospective observational data, so no causality or directionality of the effects of PTSD and physical health indicators or health behaviors can be inferred. No information was gathered about participants' possible comorbid psychiatric conditions (e.g., diagnosis of major depressive disorder), and there were numerous exclusionary criteria that may impact the external validity of this sample. Further, the questions we derived on diet content and eating behaviors were not validated with established methods of diet content assessment. The psychometric properties of the eating behaviors and diet content questions could not be examined, precluding any assessment of measurement error. Results of this preliminary analysis, however, suggest that brief, psychometrically sound measures of diet and eating behaviors in a population without eating disorders may prove helpful in determining the relationship of PTSD and engagement in health behaviors. Future studies building on the results from these preliminary data should employ validated multi-item dietary assessment instruments or more comprehensive food frequency questionnaires or eating behavior measures. Finally, this study excluded participants with self-reported eating disorders and did not assess for current or past eating disorders. Therefore, we cannot determine if the measured eating behaviors were influenced by disordered eating patterns.

In sum, we found that individuals with PTSD were significantly different from controls without PTSD across physical health indicators and engagement in both positive and negative health behaviors. Individuals with PTSD had significantly higher BMI, more alcohol use, less vigorous exercise, fewer fruit consumption, and more guilt after overeating compared to controls. These findings highlight the need for a new model of trauma, PTSD, health behaviors, and physical health indicators to examine the contributions of both unhealthy and healthful behaviors. More research is needed to comprehensively examine both positive and negative health behaviors in individuals with PTSD, especially what might account for differences in positive health behaviors like exercise, healthy diet, and eating behaviors across groups to assist in development of health behavior interventions in populations with PTSD.

## Abbreviations

AUDIT: Alcohol use disorders identification test; BMI: Body mass index; CIDI: Composite international diagnostic interview; IPAQ-SF: Short form of the international physical activity questionnaire; MANOVA: multivariate analysis of variance; METmpw: Metabolic equivalent of task minutes per week; PTSD: Posttraumatic stress disorder; UCSD: University of California San Diego; VASDHS: Veterans Affairs San Diego Healthcare System; χ2: Pearson chi-square.

## Competing interests

The authors have no competing interests to report.

## Authors’ contributions

KMG ran the analyses and prepared the manuscript. LL participated in the design of the study and was consulted on the analyses and manuscript preparation. SM ran the study and contributed to the analyses and writing of the manuscript. NA obtained funding, designed the study, consulted on the analyses, and helped prepare the manuscript. All authors read and approved the final manuscript.
